# Improving capacity for evidence-based practice in South East Asia: evaluating the role of research fellowships in the SEA-ORCHID Project

**DOI:** 10.1186/1472-6920-10-37

**Published:** 2010-05-22

**Authors:** Jacki Short, Steve McDonald, Tari Turner, Ruth Martis

**Affiliations:** 1 Previous Address: Centre for Perinatal Health Services Research, University of Sydney NSW 2006 Australia; 2 Australasian Cochrane Centre, Monash University, Victoria 3800, Australia; 3 Previous Address: Discipline of Obstetrics and Gynaecology, University of Adelaide, Women's and Children's Hospital, King William Road, North Adelaide SA 5006, Australia

## Abstract

**Background:**

Fellowships are a component of many professional education programs. They provide opportunities to develop skills and competencies in an environment where time is protected and resources and technical support are more readily available. The SEA-ORCHID fellowships program aimed to increase capacity for evidence-based practice and research synthesis, and to encourage fellows to become leaders in these areas.

**Methods:**

Fellows included doctors, nurses, midwives and librarians working in the maternal and neonatal areas of nine hospitals in South East Asia. Fellowships were undertaken in Australia and involved specific outputs related to evidence-based practice or research synthesis. Training and support was tailored according to the type of output and the fellow's experience and expertise. We evaluated the fellowships program quantitatively and qualitatively through written evaluations, interviews and follow-up of fellowship activities.

**Results:**

During 2006-07, 23 fellows from Thailand, Indonesia, Malaysia and the Philippines undertook short-term fellowships (median four weeks) in Australia. The main outputs were drafts of Cochrane systematic reviews, clinical practice guidelines and protocols for randomised trials, and training materials to support evidence-based practice. Protocols for Cochrane systematic reviews were more likely to be completed than other outcomes. The fellows identified several components that were critical to the program's overall success; these included protected time, tailored training, and access to technical expertise and resources. On returning home, fellows identified a lack of time and limited access to the internet and evidence-based resources as barriers to completing their outputs. The support of colleagues and senior staff was noted as an important enabler of progress, and research collaborators from other institutions and countries were also important sources of support.

**Conclusions:**

The SEA-ORCHID fellowships program provided protected time to work on an output which would facilitate evidence-based practice. While the fellows faced substantial barriers to completing their fellowship outputs once they returned home, these fellowships resulted in a greater understanding, enthusiasm and skills for evidence-based practice. The experience of the SEA-ORCHID fellowships program may be useful for other initiatives aiming to build capacity in evidence-based practice.

## Background

Healthcare organisations face considerable challenges in ensuring patient care is based on the best available evidence. Studies consistently demonstrate a failure to implement interventions that have been shown to be both effective and cost-effective [1]. Although this gap between evidence and practice is common to all healthcare settings, failure to bridge this gap in developing countries can have serious consequences and hinder progress towards better health [2]. Valuable resources continue to be used for practices that are out of date, have no demonstrable benefit or are even harmful, while interventions that have been shown to be both inexpensive and effective have not been widely implemented [3]. For example, it is estimated that over 70% of neonatal deaths that occur in developing countries could be prevented by the using affordable, evidence-based interventions [4].

To support evidence-based practice (EBP) change, clinicians need skills to acquire, appraise and interpret health research. There is arguably an even greater imperative for clinicians in developing country settings to have these skills as a result of the increased health burden and limited resources [5]. Critical barriers to EBP for clinicians have been well-documented and include a lack of time, insufficient resources and limited skills in accessing and applying research [6].

Fellowships are commonly used in high-income countries as a means of providing clinicians with professional training, including development of skills in research and EBP [7-12]. Fellowships offer clinicians protected time and provide access to resources and technical support that might otherwise be difficult to obtain. The practical hands-on nature of fellowships also addresses the shortcomings of training programs that rely on the provision of knowledge alone as being sufficient for skill development or practice change [13-16].

The SEA-ORCHID project (South East Asia - Optimising Reproductive and Child Health in Developing Countries) was a five-year collaborative project between four countries in South East Asia and Australia. At nine hospitals in Thailand, Malaysia, the Philippines and Indonesia, SEA-ORCHID investigated whether the health of mothers and babies can be improved through a multifaceted intervention to strengthen capacity for research synthesis, evidence-based care and knowledge implementation. The study protocol and baseline practices in perinatal health care have been published previously [17,18].

The fellowships program was one component of the intervention that also included training of clinical educators based at the nine study sites and support for a range of activities related to EBP locally and nationally. Through the fellowships, SEA-ORCHID aimed to increase capacity for EBP at participating hospitals and provide an opportunity for fellows to take time out from clinical work to gain skills and confidence to enable them to be leaders in EBP change in their professional roles.

This paper presents an evaluation of the SEA-ORCHID fellowships program, including the key results of the program in producing outputs to support EBP and clinical practice change, and the experience of the fellows involved in the program.

## Methods

### The SEA-ORCHID fellowships program

The fellowships program ran during the intervention phase of the project (2006 to 2007) and was designed to support clinicians and other professionals working at the nine SEA-ORCHID sites in South East Asia to undertake short-term EBP projects in Australia. The original research protocol specified a limited number of six-month fellowships. However, when it became clear that hospitals were reluctant to approve extended leave because of the difficulty of replacing staff and fellows themselves were often unable to be away from their families for this length of time, we decided to offer more fellowships of shorter duration.

Fellowships were tailored according to the fellows' professional background and chosen projects, the needs of their hospital, and the duration, location and timing of the fellowship. Fellows were assigned to one of the three Australian SEA-ORCHID sites (Adelaide, Melbourne, Sydney) depending on the type of support required for the fellowship and staff availability. In some cases, fellows divided their time between two Australian sites.

### Selection of fellows

The SEA-ORCHID investigators in Thailand, Malaysia, the Philippines and Indonesia selected fellows and fellowship projects on the basis of local needs for strengthening capacity in EBP. This process was pragmatic and based on subjective criteria and the availability of fellows, as well as recognising the importance of selecting fellows who were, or had the potential to be, leaders and champions for practice change [19].

Fellows were chosen from the doctors, nurses and midwives working in the maternal and neonatal areas of the nine participating hospitals. Non-clinical professionals, such as medical librarians, were not considered for fellowships in the original project proposal but their critical role in facilitating knowledge transfer to clinicians led us to reassess this and include them.

### Fellowship activities and outputs

At a broad level, fellowships were designed to equip fellows with basic knowledge and skills to support and facilitate EBP and research synthesis. This involved learning about the components of EBP (question formulation, literature searching, critical appraisal, etc.) and having the opportunity to put this knowledge into practice during the fellowship.

There was a specific structured output for each fellowship. In the fellowship proposals, fellows nominated one or more outputs to focus on during their fellowship and to continue with on their return home. The outputs were determined by each site based on local needs and perceived barriers to evidence-based care [20], and agreed to by the Australian SEA-ORCHID team. Fellowships centred on developing skills in research synthesis and supporting EBP. Planned project outputs included:

• Cochrane systematic reviews

• clinical practice guidelines

• research protocols for randomised trials

• teaching materials based on effective methods for teaching/supporting EBP and facilitating behaviour change.

Fellowships were based on adult learning principles [21] of active, self-directed, problem-based learning [22-24], critical thinking [25-27] and the provision of appropriate professional support as required. This problem-based learning approach where skills are mastered through supported practice has been a successful model of education and EBP change [28]. Formal training activities were designed in the form of interactive workshops rather than lectures as they have been found to be more likely to result in practice change [16].

Structured EBP education programs were provided which covered the concept of and need for EBP, and the skills required for EBP including asking answerable questions, database searching and critical appraisal. Training was also provided on methods of development of research protocols, systematic reviews and clinical practice guidelines as appropriate the fellowship output.

The amount of tailored training and support each fellow received depended on the type of project, the fellow's previous experience and expertise, and the time available. We also encouraged fellows to join in the routine activities of the department hosting them, including ward rounds, journal clubs, staff meetings and seminars. Wherever possible we took advantage of pre-scheduled training opportunities and enrolled fellows in Cochrane review training and research protocol-writing workshops. We also organised periods of clinical observation for the clinical fellows as we wanted them to see how clinicians incorporated EBP principles in routine clinical care.

### Evaluating the impact of the fellowships program

The evaluation of the fellowships program included:

• documenting the number and type of fellowships

• assessing the outputs of the fellowships (i.e. reviews, training materials, etc.)

• investigating the experience of fellows and barriers to and enablers of the completion of fellowship outputs.

### Data collection

The Australian SEA-ORCHID educators and project co-ordinator were responsible for collecting data from several sources for each fellow. These included written evaluations, face-to-face interviews and follow-up of fellowship activities.

#### 1) Written evaluations

Fellows completed written evaluations of the fellowship program using a structured evaluation form at the end of their fellowship and again six months later.

#### 2) Learning portfolios

Fellows were encouraged to keep a learning portfolio during their fellowship to capture thoughts and reflections about significant experiences and future plans. Although the portfolios were personal documents, fellows provided reflective summaries of these portfolios for inclusion in the evaluation of the program.

#### 3) Follow-up of outputs

Each fellow gave an update on the status of their output six months after the completion of their fellowship. For some outputs, such as Cochrane reviews, the status of the output was verified through independent sources (such as Archie, the Cochrane Collaboration's central server for managing documents). Data collection on the progress of fellowship outputs continued until July 2008.

#### 4) Interviews

Semi-structured, face-to-face interviews with the fellows were undertaken at the end of each fellowship and again after six months. The interviews were conducted by two of the Australian SEA-ORCHID educators (JS and TT) who had been closely involved in supporting the fellows. The interviews covered a range of topics, including experiences of the fellowship, how fellows had applied the knowledge and skills learned, progress with fellowship outputs, implementation plans and activities, and perceived barriers to and enablers of EBP.

### Data analysis

Data on the fellowship outputs were analysed quantitatively. Qualitative analysis included the six-month post-fellowship interviews, which were audio-recorded and transcribed by a transcription service. JS and TT reviewed and corrected the transcripts and JS analysed the data in emerging themes. NVivo software was used to store and manage the data. The written evaluations, notes from exit interviews and portfolio summaries were included in the qualitative thematic analysis.

## Results

### Fellows

SEA-ORCHID supported 23 fellowships between August 2006 and September 2007 (Table 1). There were seven fellows from Thailand, six from Malaysia and five each from the Philippines and Indonesia. The median fellowship duration was four weeks (range 2 to 13). Fellows included eight obstetricians, six neonatologists/paediatricians, five medical librarians, two labour room nurse-midwives, a neonatal nurse and a biostatistician. Three-quarters of the fellows were female.

**Table 1:** Summary of SEA-ORCHID fellowships

Country	Discipline	Location	No. of weeks	Principal activities
THA	Nurse-midwife	MEL	7	Cochrane systematic review
	Obstetrician	MEL	7	Clinical practice guideline
	Obstetrician	ADL	4	Cochrane systematic review; research protocol
	Biostatistician	MEL	13	Training materials; Cochrane review author support; Cochrane systematic review
	Paediatrician	ADL	4	Cochrane systematic review; research protocol
	Medical librarian	SYD, MEL	4	Supporting EBP, training materials
	Medical librarian	SYD, MEL	4	Supporting EBP, training materials

MYS	Neonatologist	SYD	4	Cochrane systematic review
	Neonatologist	MEL	8	Cochrane systematic review
	Nurse-midwife	ADL	4	Supporting EBP, training materials
	Medical librarian	SYD, MEL	4	Supporting EBP, training materials
	Obstetrician	ADL	2	Clinical practice guideline
	Neonatal nurse	SYD	5	Teaching and learning modules

PHI	Obstetrician	MEL	6	Clinical practice guideline
	Obstetrician	SYD, ADL	3	Cochrane systematic review
	Obstetrician	SYD, ADL	2	Cochrane systematic review
	Neonatologist	MEL	4	Clinical practice guideline
	Medical librarian	SYD, MEL	4	Supporting EBP, training materials

IND	Neonatologist	MEL	4	Cochrane systematic review
	Neonatologist	MEL	3	Cochrane systematic review
	Neonatologist	MEL	4	Cochrane systematic review
	Obstetrician	MEL	4	Cochrane systematic review
	Medical librarian	SYD, MEL	4	Supporting EBP, training materials

### Fellowship Outputs

The main outputs of the fellowships were drafts of Cochrane systematic reviews, clinical practice guidelines (CPGs) and protocols for randomised controlled trials (RCTs), and the development of training materials to support EBP (see Tables 2, 3, 4, 5 below). At the end of follow-up in July 2008, five fellows had published and six had submitted protocols for Cochrane systematic reviews, with one still in the draft stage. Five fellows had early drafts of CPGs and three had drafts of research protocols.

**Table 2:** SEA-ORCHID fellowships - systematic review topics

Protocols for Cochrane systematic reviews	Status as at July 2008
Calcium supplementation (other than for preventing or treating hypertension) for improving pregnancy and infant outcomes	Published
Dilute versus full strength preterm formula for preterm or very low birth weight infants	Published
Skin preparation for preventing infection during caesarean section	Published
Anticoagulant therapy for deep vein thrombosis (DVT) in pregnancy	Submitted
Techniques of monitoring blood glucose in women with gestational diabetes	Submitted
Different antibiotic regimens for the treatment of asymptomatic bacteriuria in pregnancy	Submitted
Relaxation therapy for preventing preterm labour	Published
Specialty teams for neonatal transport to tertiary centres	Published
Varying proportion of non-protein energy provided as lipids for parentally fed neonates	Submitted
Folic acid supplementation for the prevention of anemia in premature neonates	Submitted
Calcium and phosphorus supplementation of formula milk for preterm infants	Submitted
Vitamin D supplementation for prevention of morbidity and mortality in preterm infants	Draft

**Table 3:** SEA-ORCHID fellowships - guideline topics

Clinical practice guidelines	Status as at July 2008
Prevention of hospital-acquired infections among newborns	Formation of steering group
Corticosteroids in women at high risk of preterm birth	Very early draft.
Management of pre-eclampsia	Very early draft.
Management of endometritis	Very early draft.
Continuous positive airway pressure for newborn care	Very early draft.

**Table 4:** SEA-ORCHID fellowships - RCT topics

Research protocols for randomised trials	Status as at July 2008
Single dose cefazolin for preventing post-surgical infection in miscarriage	Draft
Vitamin D supplementation for prevention of vitamin D deficiency and its complications in preterm infants ≤ 37 weeks	Draft
Antenatal probiotic supplementation for preventing preterm birth	Draft

**Table 5:** SEA-ORCHID fellowships - Other outputs

Other fellowship outputs	Status as at July 2008
RHL commentary suturing techniques for 3^rd ^and 4^th ^degree tears	Published
RevMan 5 tutorial, Thai training materials/exercises	Completed
Teaching modules on CPAP and pain management in neonates	Completed and routinely incorporated into staff training workshops
Teaching modules on accessing evidence-based health information	Completed and implemented in each participating site

Six months after completion of each fellow's program there had been some development in the drafting and submission process for Cochrane systematic reviews but little progress on development of RCTs or CPGs.

Twelve of the fellows undertook a Cochrane systematic review and registered their topic with either the Cochrane Pregnancy and Childbirth Group or Cochrane Neonatal Group. By the end of the fellowship most fellows had a protocol for their review that was either an advanced draft or had been submitted to the respective review group. By July 2008 all but one protocol had been submitted, two had been published in *The Cochrane Library *and the remainder were in various stages of editorial review. Fewer fellows attempted to tackle clinical practice guidelines or RCT protocols, and their progress was slower.

Fellows also reported that there was considerable activity in delivery and dissemination of EBP training (especially arising from the fellowships for medical librarians), and an increase in evidence-based clinical practice change, journal clubs and Cochrane Library use.

Most of the fellows were clinicians who, in addition to the research and education components of their fellowships, reflected on the implications of EBP for their clinical work. As a result, many of the fellows were keen to implement specific clinical practice changes which they had often observed in Australia. In this way the fellowships will directly contribute to the impact of the SEA-ORCHID project on healthcare processes and outcomes.

### Fellows' experience

#### Benefits of the fellowships

Overall the fellows reported that they enjoyed the fellowships program and gained skills and experience in EBP and research use that were beneficial for them as well as their hospitals.

*I had an immensely beneficial time on this fellowship, obtaining invaluable hands-on experience in preparation of a systematic review and utilising available evidence to improve clinical care*.

*After finishing my Cochrane systematic review, I also can apply the results into my clinical practice and be a role model and mentor for the residents and medical students to help them develop a Cochrane systematic review in the future*.

Fellows also reported gaining skills and confidence in areas beyond the confines of their specific fellowship output.

*Before I came to Australia, I only had some experience in conducting RCTs and Cochrane systemic review but no experience with CPG appraisal, adaptation and development with the AGREE tool. On this fellowship training program many perfect resources were provided to help with the topics above. This has made me more confident to conduct a quality RCT, develop or appraise a CPG and be more skilful in searching for evidence in the literature to resolve a clinical problem*.

Some fellows noted that the experience of the fellowship, even without completing their specific project, had enabled them to change clinical practice.

*The systematic review is still in the process of being completed. However, the information gained during the literature search performed has enabled certain changes be made with regards to management of folic acid supplementation in premature newborns*.

Other fellows reported making changes in a range of areas including encouraging mobility during first stage of labour, allowing fluid and food during early labour, prevention of neonatal infection and changes in use of birthing positions.

Several fellows noted other career or personal benefits resulting from the fellowships program, such as gaining promotions.

*I think the [fellowship] helped getting my promotion. ... I have more confidence to speak up and to show my interest to the interviewer regarding my profession*.

The fellows identified that the components critical to the success of the fellowships program were having protected time, access to academic expertise and resources, tailored training, opportunities for clinically relevant research and opportunities to present and get feedback on their work.

#### Barriers to completing outputs

Fellows identified several barriers to successfully completing their fellowship outputs once they returned home. These included lack of time, lack of equipment and lack of access to the internet and evidence-based resources such as *The Cochrane Library*.

*Because for me when I went back to [my country], when I am trying to continue my fellowship, what I have done in the fellowship time is very difficult, because of the time; I have very, very tight time in here*.

*Maybe first when we were [in Australia], the work is very easy because you were there and the things were there and the computer access is very good, but when we came here [home] the internet access ability is very low. When we can work in Australia for one day, maybe we have to do it here for three days to one week*.

#### Enablers of completing outputs

Fellows identified a range of enablers of the completion of fellowship outputs. Access to computers and evidence-based internet sources to overcome the barriers identified above was regarded as an important enabler.

Support from senior staff, colleagues and junior staff at their home institution was also noted as an important enabler of progress on the fellowship output. External research collaborators from other institutions and other countries were recognised as another important source of support.

*The support we get [from SEA-ORCHID] helps us get our researches finished and published*.

*[SEA-ORCHID Investigator] has started research groups with membership from different institutions. The members may function as support groups to each other in conducting research and implementing evidence-based practice*.

Fellows recognised the importance of protected time in enabling completion of their fellowship outputs, but were not able to identify how this could be secured once they returned home.

## Discussion

The SEA-ORCHID fellowships program resulted in 23 fellows, from a range of backgrounds and with diverse skills and experience, producing evidence-based training materials, drafts of Cochrane systematic reviews, clinical practice guidelines and trial protocols, plans for clinical practice change and a commentary for the WHO Reproductive Health Library. Six months after completing their fellowships there was some development in the drafting and submission process for Cochrane systematic reviews but little development of RCTs or CPGs. There was considerable activity in the delivery and dissemination of EBP training and materials, reported clinical practice changes, journal clubs and Cochrane Library use.

The benefits of the SEA-ORCHID fellowships program to both fellows and the hospitals from which they were selected are similar to those reported from clinical fellowship programs in high-income countries like Australia and the United States [10,11]. Most fellows made substantial progress towards their chosen output and all gained substantial skills and experience in the elements of EBP. Several fellows reported that they were able to make practice changes in their hospitals and some also identified personal accomplishments resulting from the fellowship.

One of the main outputs of the fellowships program was the production of Cochrane systematic reviews, designed to be a practical way of introducing clinicians to the links between clinical practice and research. Systematic reviews and guidelines can take years to complete, so not surprisingly, busy clinicians returning home struggled to complete protocols for their reviews. Within six months of returning to busy clinical practices, and without protected time, it was not feasible for some fellows to complete even the protocol for their reviews.

While we did not expect these outputs to be completed within the fellowship period or the six month follow-up, we hoped that the fellowships would enable greater understanding of, skill in and access to resources necessary for EBP, and this appears to have happened.

The importance of selecting fellows who were practicing clinicians and librarians (and able to directly translate knowledge into practice change) cannot be overstated, and this has also been identified by other studies [10]. While knowledge champions can drive practice change, the perception and/or reality of limited power in a workplace can impede this [29]. In this project, fellows' roles as senior clinicians, librarians and educators in their hospital departments and universities meant they could strongly influence the teaching and practice of junior staff and students. Both the number of evidence-based training modules and materials developed during the fellowships and the amount of dissemination activity post-fellowship, suggest that this area of output of the fellowships was very successful. The program also highlighted the critical role medical librarians play in knowledge translation and their participation was particularly valuable.

This study has some limitations. There were some difficulties with data collection which make definitive conclusions difficult to draw. It was not always possible to measure progress with fellowship outputs. For instance, in the case of some training workshops, it was not always clear what was being taught and how, with whom, for how many, how often and what the outcomes were (i.e. changes in knowledge, skills and practice). Sometimes it was unclear if the fellowship output was an entirely new activity or how it extended or enhanced existing EBP activities. It was also not always clear at what stage drafts of SRs, RCTs, and CPGs were up to or whether fellows still plan to complete them. Fellowship evaluation interviews were conducted by fellowship hosts which may have compromised impartiality, however data collection, analysis and interpretation were undertaken in awareness of this.

Protected time, access to academic/technical expertise and resources, training, opportunities for clinically relevant research and opportunities for presentation of and feedback on work were all identified as critical components to the success of the fellowships program. Some suggestions for improving the program included: stronger support for links between fellows after their return from the fellowships; continuing to build and foster relationships with external collaborators; provision of access to well maintained computers, the internet and EBP resources in the fellows' work environments.

The capacity of the fellows selected for the program is also a critical success factor. The motivation and commitment of fellows to spend time in another country (in some cases for the first time) and work on a project (often in a second language) took remarkable courage and perseverance. Acknowledging the achievements of the 23 fellows, in often very difficult circumstances, is vital to ensure the gains from these fellowships continue. During the six-month follow-up data collection, some fellows were embarrassed and apologetic about not having been able to complete outputs as they might have hoped. Despite significant environmental challenges, the personal and professional commitment of fellows to bring about change in their hospitals and universities is a tribute to the fellows.

The results of this study highlight the potential value of fellowship programs as a method of increasing capacity in EBP. They also highlight key barriers that need to be addressed for fellowships to be ultimately successful and enablers that can be used to achieve this aim. These factors should, therefore, be considered when designing fellowship programs and selecting fellows for future capacity building initiatives.

## Conclusions

The SEA-ORCHID fellowships program provided protected time for fellows to work on individually selected, clinically relevant, projects which would produce specific outputs and lead to greater implementation of EBP. While the fellows faced substantial barriers to completing their project outputs once they returned to their clinical environments, these fellowships resulted in a greater understanding of, skill in, and enthusiasm for EBP. The experience of the SEA-ORCHID fellowships program may be useful for other initiatives aiming to build capacity in evidence-based practice.

## Competing interests

The authors declare that they have no competing interests.

## Authors' contributions

JS, SM, RM and TT implemented the fellowship program, and supported and trained the fellows. JS and TT undertook the interviews. JS collected and analysed the data and prepared the first draft of the article. SM and TT refined the draft. JS, SM, RM and TT approved the final manuscript.

## Pre-publication history

The pre-publication history for this paper can be accessed here:

http://www.biomedcentral.com/1472-6920/10/37/prepub
